# Unlocking a closed system: dosimetric commissioning of a ring gantry linear accelerator in a multivendor environment

**DOI:** 10.1002/acm2.13116

**Published:** 2021-01-15

**Authors:** Amarjit Saini, Chris Tichacek, William Johansson, Gage Redler, Geoffrey Zhang, Eduardo G. Moros, Muqeem Qayyum, Vladimir Feygelman

**Affiliations:** ^1^ Department of Radiation Oncology Moffitt Cancer Center Tampa FL USA; ^2^ RaySearch Americas Inc New York NY USA

**Keywords:** IMRT/VMAT verification, MLC modeling, TPS beam modeling

## Abstract

The Halcyon™ platform is self‐contained, combining a treatment planning (Eclipse) system TPS) with information management and radiation delivery components. The standard TPS beam model is configured and locked down by the vendor. A portal dosimetry‐based system for patient‐specific QA (PSQA) is also included. While ensuring consistency across the user base, this closed model may not be optimal for every department. We set out to commission independent TPS (RayStation 9B, RaySearch Laboratories) and PSQA (PerFraction, Sun Nuclear Corp.) systems for use with the Halcyon linac. The output factors and PDDs for very small fields (0.5 × 0.5 cm^2^) were collected to augment the standard Varian dataset. The MLC leaf‐end parameters were estimated based on the various static and dynamic tests with simple model fields and honed by minimizing the mean and standard deviation of dose difference between the ion chamber measurements and RayStation Monte Carlo calculations for 15 VMAT and IMRT test plans. Two chamber measurements were taken per plan, in the high (isocenter) and lower dose regions. The ratio of low to high doses ranged from 0.4 to 0.8. All percent dose differences were expressed relative to the local dose. The mean error was 0.0 ± 1.1% (TG119‐style confidence limit ± 2%). Gamma analysis with the helical diode array using the standard 3%Global/2mm criteria resulted in the average passing rate of 99.3 ± 0.5% (confidence limit 98.3%–100%). The average local dose error for all detectors across all plans was 0.2% ± 5.3%. The ion chamber results compared favorably with our recalculation with Eclipse and PerFraction, as well as with several published Eclipse reports. Dose distribution gamma analysis comparisons between RayStation and PerFraction with 2%Local/2mm criteria yielded an average passing rate of 98.5% ± 0.8% (confidence limit 96.9%–100%). It is feasible to use the Halcyon accelerator with independent planning and verification systems without sacrificing dosimetric accuracy.

## 1 INTRODUCTION

The Halcyon™ radiotherapy platform (Varian Medical Systems, Palo Alto, CA, USA) is designed as a self‐contained system, combining an integrated treatment planning system (TPS), information management/Record and Verify (R&V) system and a ring‐gantry radiation delivery device. The beam model in the integrated TPS (Eclipse) is provided and locked down by the vendor. The machine is essentially tuned to match a set of predefined beam specifications, including the percentage depth doses (PDDs) and cross‐beam profiles. The rest of the dosimetric commissioning process is simply validation of the noneditable TPS model. A portal dosimetry‐based system for patient‐specific dosimetric QA (PSQA) is also a part of the preconfigured package. The tests of the entire system and its components have been thoroughly described in the literature, for both prototype and clinical systems.^1^, ^2^, ^3^, ^4^

While the advantages of the preconfigured system in terms of uniformity across the user base are undeniable, this approach may not always be optimal in practice. Case in point is implementation in our large radiotherapy department with over 50 active TPS users. A new TPS has been recently installed and commissioned as the primary planning system for the majority the linear accelerators in the department. Providing an adequate number of planning licenses on a Halcyon‐specific TPS, as well as training and maintenance, for a single accelerator would constitute a non‐trivial financial and human resource burden. Consequently, we set out to explore if it was possible to leverage the existing TPS capabilities to plan for the Halcyon accelerator without compromising accuracy. In addition, an independent semiempirical system for PSQA was also validated for use with Halcyon since it is already in use throughout the department.

## 2 MATERIALS AND METHODS

### 2.1 Treatment planning and delivery

The Halcyon (v. 2.0) is configured with a single 6 MV flattening filter free (FFF) beam (nominal dose rate 800 cGy/min at d_max_ of 1.3 cm). It is equipped with a stacked‐and‐staggered dual‐layer MLC.^3^ While each leaf casts a 1 cm shadow at isocenter (100 cm from the source), the staggered design results in effective leaf with of 0.5 cm. The MLC is single focused (in‐plane) and the leaves have rounded ends. The curvature radius (23.4 cm) is larger than for the standard (8 cm) or high‐definition (16 cm) Varian 120‐leaf MLCs.^3^ Leaf height is 7.7 cm vs 6.5 to 6.75 cm for 120‐leaf collimators (depending on the model and leaf position in the bank), reducing leaf transmission.^3^ The maximum field size at isocenter is 28 × 28 cm^2^. Machine scales are fully compliant with International Electrotechnical Commission (IEC) Standard 61217.

Eclipse shares the database with Aria v. 15.6.7 R&V system (Varian) which in turn passes the information to the accelerator. For commissioning purposes, the R&V system can be bypassed in the Service mode and DICOM RT Plan files loaded directly through the accelerator console.

The primary TPS investigated in this study was RayStation v.9B (RaySearch Laboratories, Stockholm, Sweden). It fully supports the Halcyon geometry, including the double‐stacked and staggered MLC configuration, and dose delivery parameters. As with the rest of our machines, we commissioned only the Monte Carlo (MC) dose calculation engine. For comparison to Eclipse, the identical plans were recalculated with the vendor‐configured TPS supplied with the Halcyon system, using the grid‐based Boltzmann equation solver (ACUROS™)^5^ algorithm v. 15.6.06.

### 2.2 Experimental data collection

#### 2.2.1 The roadmap

The dosimetric commissioning process followed these general steps. The cross‐beam profiles and PDDs were collected in a water tank with different detectors and used for the beam energy and profile modeling. Relative output factors were collected with appropriate detectors to establish output correction factors in the beam model. Specials measurements were taken to help with MLC modeling. Those included dynamic fields measurements with a Farmer chamber following the dosimetric leaf gap (DLG) concept^6^, ^7^ and various static abutting fields scanned with a small diode. Finally, a set of modulated plans was used to hone the model against multiple ion chamber measurements and additionally verify it with a diode array.

#### 2.2.2 Beam profiles and PDDs

The MC algorithm implementation in RayStation follows a mixed approach. The accelerator head is not simulated, but rather the phase space above the moving parts is deduced form the experimental dose distributions in water.^8^ The resulting fluence is modulated by the transmission values of the MLC leaves (Halcyon has no movable jaws). The MC simulation starts downstream once the fluence encounters the patient boundary described by the external contour. To establish the phase space, a set of beam profiles and PDDs was collected. In addition to verifying the machine‐specific dose distribution, the goal was to compare the ion chamber and diode scans for the Halcyon beam and specific detectors, to optimize the commissioning process. The detailed description of the collected data and instrumentation is tabulated in the Appendix.

PDDs for the fields ≤10 × 10 cm^2^ were indistinguishable between the Edge diode (Sun Nuclear Corp. Melbourne, FL, USA) and CC13 IC (IBA, Schwarzenbruck, Germany). For the fields larger than 10 × 10 cm^2^, cross‐beam profiles obtained with the diode were used for MLC leaf end modeling while the IC data helped with adjusting the beam shape well within the field. The CC13 IC and Edge diagonal profiles for a 28 × 28 cm^2^ field showed a small but consistently noticeable difference away from the central axis (about 1%).

For the step‐and‐integrate small field PDD measurements, the search for the maximum signal was performed at d_max_ and every 5 cm in depth thereafter. The Halcyon MLC geometry does not allow for a centered 0.5 × 0.5 cm^2^ field. However, it is possible to construct such a field with the center shifted 0.25 mm perpendicular to the leaf movement direction. The profiles and PDDs were intercompared between the standard beam data, our measurements with two detectors, and RayStation calculations. These comparisons included one‐dimensional gamma analysis with the open source “MPPG 5a tool.”^9^

#### 2.2.3 Relative output factors

In RayStation, introduction of a relative output value for a particular field size is possible only if a corresponding depth‐dose curve is also present. Collecting the small‐field PDDs allowed us to include small‐field values in the output optimization process. Among the three detectors used the IC and water‐equivalent scintillator (W1, Standard Imaging Inc., Middleton, WI, USA) require no field size corrections in the respective ranges of field sizes.^10^, ^11^ For the semiconductor Edge detector, we used Monte Carlo‐based field size corrections derived for a 6 MV FFF beam at ViewRay Inc. (private communication). Our previous experiments confirmed agreement between the Edge (with those corrections) and scintillator measurements to within ≤1% for the square field sizes down to 0.415 cm on a side. The detector was centered in the field by searching for the maximum signal in two dimensions. The Edge and CC13 IC were horizontal, while the W1 was positioned with the long axis toward the source (i.e., 1 mm collecting volume diameter and 3 mm length). The output data were compared against the RayStation calculations. Here and elsewhere the MC calculation statistical uncertainty (one standard deviation averaged between all voxels receiving ≥50% of the maximum dose) was set to 0.3%. For the smallest fields (≤2 × 2 cm^2^) the central voxel dose was taken as the RayStation output. The dose grid was 1 × 1 × 3 mm^3^. For the rest of the fields, an isotropic 2 mm grid was used and the mean dose to a small cylindrical region of interest approximating a CC13 chamber represented the calculated output.

#### 2.2.4 Data for fine‐tuning MLC parameters

*Leaf edge (Tongue‐and‐Groove (T&G) width*. Separately for the distal and proximal banks, complementary bar patterns were created with the open leaves in one replacing the closed leaves in the other. Each pattern was scanned with the Edge detector in the Y (in‐plane) direction. By adding the two diode scans together and comparing to the RayStation‐calculated profile, the T&G width can be evaluated and adjusted if necessary.

*Leaf end (MLC offset)*. The data collected in this section included both static and dynamic measurements carried out at the central axis and shifted across the field in the leaf travel direction.^6^ The field edge positions (as defined by the inflection point on the penumbra profile of an FFF beam ^12^ determined by the extremum of the first derivative of the penumbra profile) were extracted from the diode profiles to assess the linearity of MLC positioning across the field. Another static measurement was the abutting fields test, which is quite sensitive to the MLC end modeling parameters. Two abutting 2 × 4 cm^2^ (IEC X × Y) fields were separately scanned in the X direction at the depth of 10 cm with the Edge detector. The apertures were defined by both leaf layers. Care was taken to always scan in the same direction to minimize the positional uncertainty. The scanning curves were normalized to the center of the respective openings and summed. The data from three runs were averaged. The summary profile was compared to the high‐resolution calculations (1 mm^3^ voxel) in RayStation. This procedure was performed with the abutment line at 0 and ±10 cm X positions.

Another approach to the leaf‐end modeling is dynamic measurements involving MLC‐defined gaps sweeping across the field. The first version was the classic dosimetric leaf gap (DLG) measurement.^6^, ^7^ The variable sweeping gaps (2–20 mm wide) measurements with a Farmer chamber were carried out in a Plastic Water phantom at 10 cm depth and 90 cm SSD. They were done at X = 0, ±3, 5, 10, 12, and 13 cm. The chamber signal minus the leakage was plotted against the gap width and the negative of the x‐axis intercept of the linear fit line defined the DLG. Alternatively, we converted the chamber readings in the same setup into absolute dose by cross‐calibrating against the RayStation calculation in the 10 × 10 cm^2^ static field at 10 cm depth and plotted the difference with the RayStation calculations for different gap widths. With this approach, leakage is not subtracted from the chamber reading since it is inherent in the calculations. The RayStation calculations were performed with varying MLC offset parameters to optimize those. The goodness‐of‐fit metric was the root‐mean‐square (RMS) difference between the measured and calculated doses to the Farmer chamber across all gap sizes.

#### 2.2.5 MLC parameters in the RayStation model

Ideally, the algorithm should ray trace through the MLC modeled with correct dimensions and density to arrive at the fluence downstream, as demonstrated by Losasso et al in 1998.^7^ However, the MLC model in RayStation employs significant simplifications. The leaf has no height but is rather treated as an infinitely thin object with user‐specified transmission.^13^, ^14^ At the tip, a small area with transmission increased from T to $\sqrt{T}$ (as if the leaf height there was ½ of its normal value) is added (“MLC tip width”). It is meant to account for the rounded leaf end. Various parameters are interrelated in how they affect the fluence downstream of the MLC and multiple combinations can lead to approximately the same dosimetric results, although the parameters can be treated as independent for modeling purposes.^15^, ^16^ Our strategy was to optimize and fix the values that can be easily derived from the isolating experiments first, and then fine‐tune the rest for best agreement with dose measurements with modulated test plans.

*Distance from the source*. As the MLC leaves have no thickness in RayStation, their distance from the source in the model is somewhat arbitrary, that is, the same geometric penumbra values can be achieved for different combinations of distance and source size. Also, even though the proximal and distal layers technically have individual distances specified, only the value for the upper one is used in the final dose calculation. A sensible approach was to place the proximal and distal MLCs at the corresponding bottom‐of‐the leaf distances, 38.9 and 47.9 cm, respectively.^17^ This way approximately the mid‐point between the two layers was used as the source‐to‐leaf distance for calculations.

*MLC transmission*. With Millennium MLCs, leaf transmission is often used as a catch‐all parameter to fine‐tune the final dosimetric agreement for modulated beams. However, the transmission through the double‐stacked Halcyon collimator is nearly negligible and varying it would not help much with changing the calculated IMRT/VMAT dose. Therefore, the best strategy is to settle at the measured/standard transmission values which were reported to be of the order of 0.4 ‐ 0.5% for individual layers and nearly negligible for the fully stacked configuration.^2^, ^3^ The transmission values were confirmed with a Farmer chamber measurements at 10 cm depth and the standard Eclipse value of 0.47% was deemed sufficiently accurate.

*MLC tip width*. Except for the very recent paper that takes a unified approach to the MLC parameter optimization,^14^ at the time of this work the recommendation in the literature was to compare measured static beam profiles with calculations employing different leaf tip width values.^13^ That matched our experience with commissioning the Millennium MLCs in RayStation. While the height of the central peak at the field junction line is primarily determined by the leaf offset, the leaf tip width influences the shape of the flatter portions of the dose profiles adjacent to the peak. Combining simple geometrical considerations with published optimization results for the Millennium collimators,^14^ the expected field tip width should not exceed ~0.1 cm. The shape of the abutting fields profiles was compared for the field tip widths of 0.0 and 0.1 cm, with all other offset parameters set to 0. Generally, the leaf tip width influences the final results less than the leaf offset,^16^, ^18^ particularly with the lower transmission of the Halcyon MLC compared to the Millennium.

*MLC Offset*. This is probably the most important part of MLC modeling, greatly affecting the final dosimetric results. MLC tip positions $X_{\mathrm{RS}}$ used for calculation of the projections in RayStation are governed by Eqs. (1) and (2) for the left and right MLC banks, respectively.^8^, ^14^

(1) $$X_{\mathrm{RSLt}}=X_{\mathrm{nom}}‐Offset+Gain\Delta X_{\mathrm{nom}}‐Curvature\Delta X_{\mathrm{nom}}^{2}$$

(2) $$X_{\mathrm{RSRt}}=X_{\mathrm{nom}}+Offset+Gain\Delta X_{\mathrm{nom}}+Curvature\Delta X_{\mathrm{nom}}^{2}$$

where $X_{\mathrm{nom}}$ is the nominal leaf position and *Offset*, *Gain*, and *Curvature* are the MLC parameters in the RayStation Fluence tab. The X coordinate is positive for both left and right leaf banks to the right of the central axis and is negative to the left of it, as seen in beam’s eye view.

The analysis of these equations is best carried out with introduction of two more variables. For a pair of opposing leaves both with the nominal planned position $X_{\mathrm{nom}}$, the dosimetric offset, $\Delta X_{D}=\left(X_{RSRt}‐X_{RSLt}\right)/2$, accounts for the difference between the dosimetric field edge in RayStation and the nominal leaf position. The shift of the calculated midpoint position between the leaves and the nominal position is defined as $\Delta X_{\mathrm{MP}}=\left(X_{RSLt}+X_{RSRt}\right)/2‐X_{\mathrm{nom}}$.

The leaf offset equations have some interesting practical properties. *Offset* and *Curvature* affect the $\Delta X_{D}$but not the $\Delta X_{\mathrm{MP}}$. Conversely, *Gain* affects the $\Delta X_{\mathrm{MP}}$but not the $\Delta X_{D}$. Thus, the following strategy for optimizing the MLC parameters was adopted. First, adjust *Gain* to maintain, as close as possible on average, the distance between the calculated and measured open field profile edges for different field widths. Then adjust *Offset* and, if necessary, *Curvature* to obtain the best dosimetric fit using the combination of static and dynamic offset measurements described above. The *Curvature* parameter modifies $\Delta X_{D}$off‐axis. Finally hone these parameters based on the measurements of the highly modulated IMRT/VMAT test plans.

*The kinematic and other delivery parameters* were taken from the published values and the Eclipse configuration data. The MLC speed was set at 5 cm/s and gantry rotational speed at 12⁰/s (2 rpm). The dynamic leaf gap was set conservatively at 0.06 cm vs 0.05 cm in Eclipse to prevent the effect of possible roundoff errors. Notably, the minimum MU/deg value had to be set at 0.1 MU/deg to avoid undeliverable beams, contrary to the Eclipse parameter page (0–60 MU/deg).

### 2.3 IMRT/VMAT tests

#### 2.3.1 Ion chamber measurements

The test plans in RayStation were developed for five cases — C‐shape (easier) from TG119^19^ and four datasets from MPPG 5a^20^ — Head and Neck, Prostate Bed, Abdomen, and Anal. The target contours on two of the latter four were modified to fit in the 28 × 28 cm^2^ maximum Halcyon field size. Three plans were developed for each dataset: traditional VMAT, sliding window VMAT (with all leaves sweeping the field in one direction at any given time),^8^ and sliding window IMRT with nine equidistant gantry angles. The phantom plans were later transferred to Eclipse for recalculation and comparison, preserving the phantom material (water), density overrides, and the dose grid size (isotropic 2 mm voxels).

Point dose (ion chamber) measurements were performed in a 30 × 30 × 15 cm^3^ plastic phantom. The phantom was represented in the TPS as water with unity density. Two Model 31010 0.125 cc (PTW, Freiburg, Germany) ICs per plan were placed in suitable locations on a 5 × 5 grid pattern in the central 12 × 12 cm^2^ area of the phantom. All locations had the center of the collecting volume residing in the same transversal plane aligned to the machine isocenter. The chambers’ daily correction factors were obtained by cross‐calibration to RayStation dose at isocenter in the phantom in the parallel‐opposed 10 × 10 cm^2^ fields. Whenever feasible, the VMAT/IMRT plans had additional objectives to make the dose in a small volume surrounding the chambers’ locations as homogeneous as possible. One chamber was placed in the high‐dose region (at the isocenter) and another in the lower dose location. The low to high dose ratios ranged from 0.4 to 0.8. All percent dose errors were reported normalized to the *local* dose, which is an unbiased and stringent measure of the algorithm accuracy.

#### 2.3.2 Diode array measurements

Dose‐distribution measurements were performed with a well‐characterized helical diode array — the ArcCHECK (Sun Nuclear)^21^, ^22^, ^23^, ^24^ with SNC patient software v. 8.3. The phantom was represented in the TPS as a uniform PMMA cylinder with density of 1.19 g/cm^3^. The dosimeter was cross‐calibrated daily in the parallel opposed fields to minimize differences in the central portion of a corresponding RayStation plan. The results of the gamma analysis comparison were reported with the standard 3% dose error with global normalization /2 mm distance to agreement criteria (3%G/2mm),^25^ as well as with 3 and 2% local (L) dose error. The minimal dose threshold was always kept at 10%. In addition, the local dose errors per detector were extracted and analyzed outside of the SNC software.

All statistical analyses were done in GraphPad Prizm software package v.8 (GraphPad Software, San Diego, CA, USA). Confidence level of 95% was considered statistically significant, unless otherwise stated.

#### 2.3.3 Independent patient‐specific dose verification method

Halcyon offers a built‐in portal dosimetry patient‐specific verification method. However, our standard method for patient‐specific dosimetric quality assurance is semiempirical 3D dose reconstruction (PerFRACTION [PF] v. 3.0.2, Sun Nuclear),^26^ which we intended to validate for the use with Halcyon. The plan is delivered prior to the first fraction with nothing in the beam path, and dose is recalculated on the patient dataset using a new DICOM RT PLAN object, wherein all possible control point parameters are harvested from the accelerator log (trajectory) files.^26^, ^27^, ^28^ Additionally, if time‐resolved (cine) EPID images are available, the log files MLC positions are replaced with those extracted from the EPID movie. With Halcyon machines, there is no cine EPID option and the log file data alone have to be used. As is the case with Eclipse, the PF Halcyon beam model is based on the standard dataset and customization is not offered. The IMRT/VMAT calculations were repeated with the PF’s convolution/superposition dose engine (DoseCHECK)^27^, ^28^ on the cube phantom, and point doses compared to the IC results. The volumetric dose distributions were also compared between RayStation and PF by gamma analysis with 3%G/2 mm and 2%L/2 mm criteria combinations. The phantom material was again treated as water to focus on the beam/MLC models as opposed to differences in heterogeneity handling by different algorithm families and implementations.

## 3 RESULTS

### 3.1 Beam profiles and PDDs

Representative cross‐beam profiles and PDDs were compared to our water scans with gamma analysis using 2%L/1 mm criteria for the 2 × 2, 10 × 10 and 28 × 28 cm^2^ fields. The cutoff thresholds were adjusted per field size (3 to 5%) to include the outside toe of the penumbra but exclude the noisy low‐dose regions outside the primary beam that would otherwise skew the results with local dose‐error normalization. The lower passing rate for the 2 × 2 cm^2^ PDD (91%) is attributable to the >2% disagreement at the shallow and >20 cm depths (the PDD curves in RayStation are normalized to agree with the output at 10 cm depth). The reported agreement at the deeper end of the curve would be better than 2% if the often‐quoted global (Van Dyk)^29^ dose‐error normalization was used. The PDD gamma analysis passing rates for the two larger fields are above 99%. The average of passing rates for aligned profiles at four depths for all field sizes plus diagonal ones for the largest field was 98.4 ± 2.1% (1SD) with the range 93.3%–100%.

**Fig. 1:**
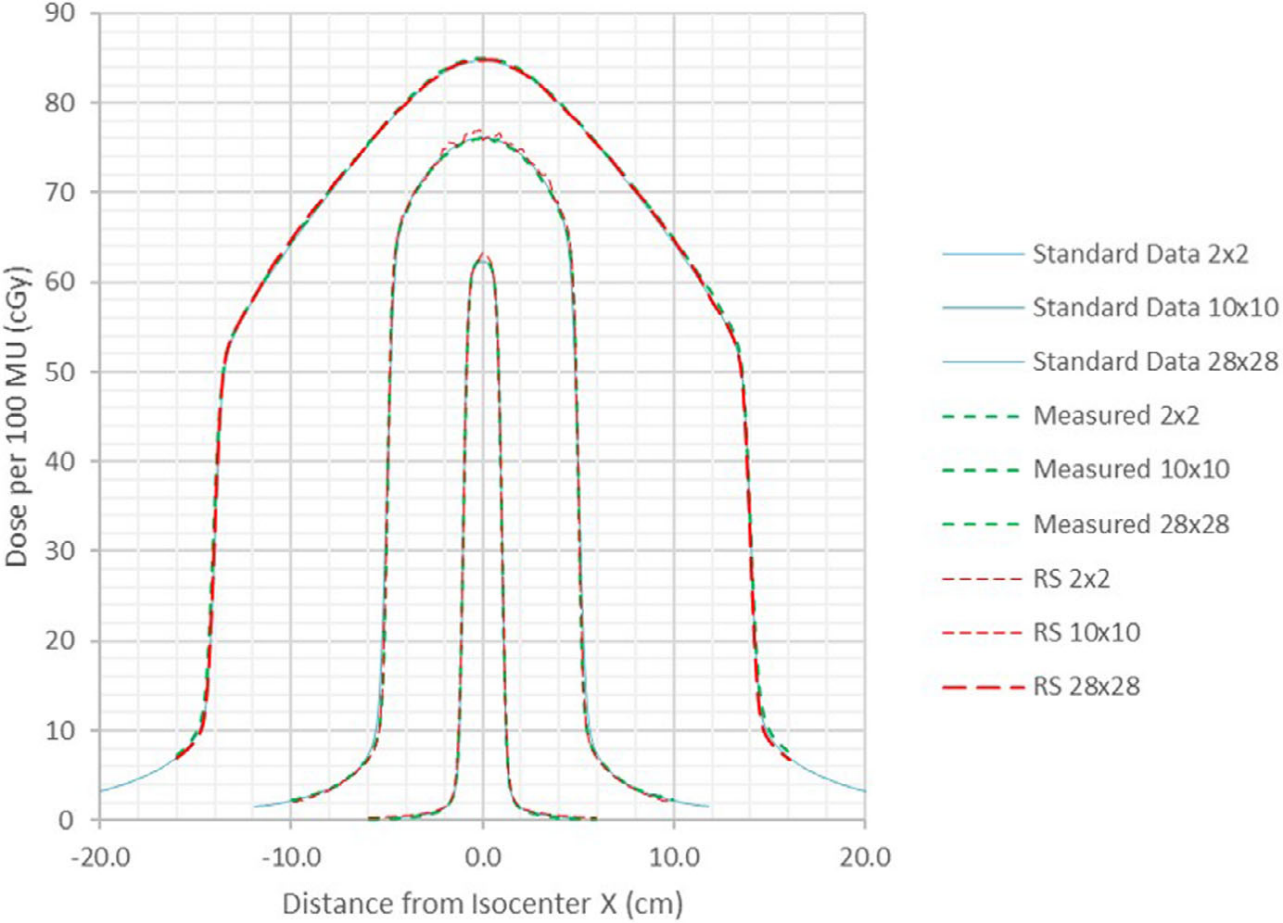
Overlaid cross‐plane profiles at 10 cm depth from standard Varian Halcyon data, our measurements, and RayStation calculations.

**Fig. 2:**
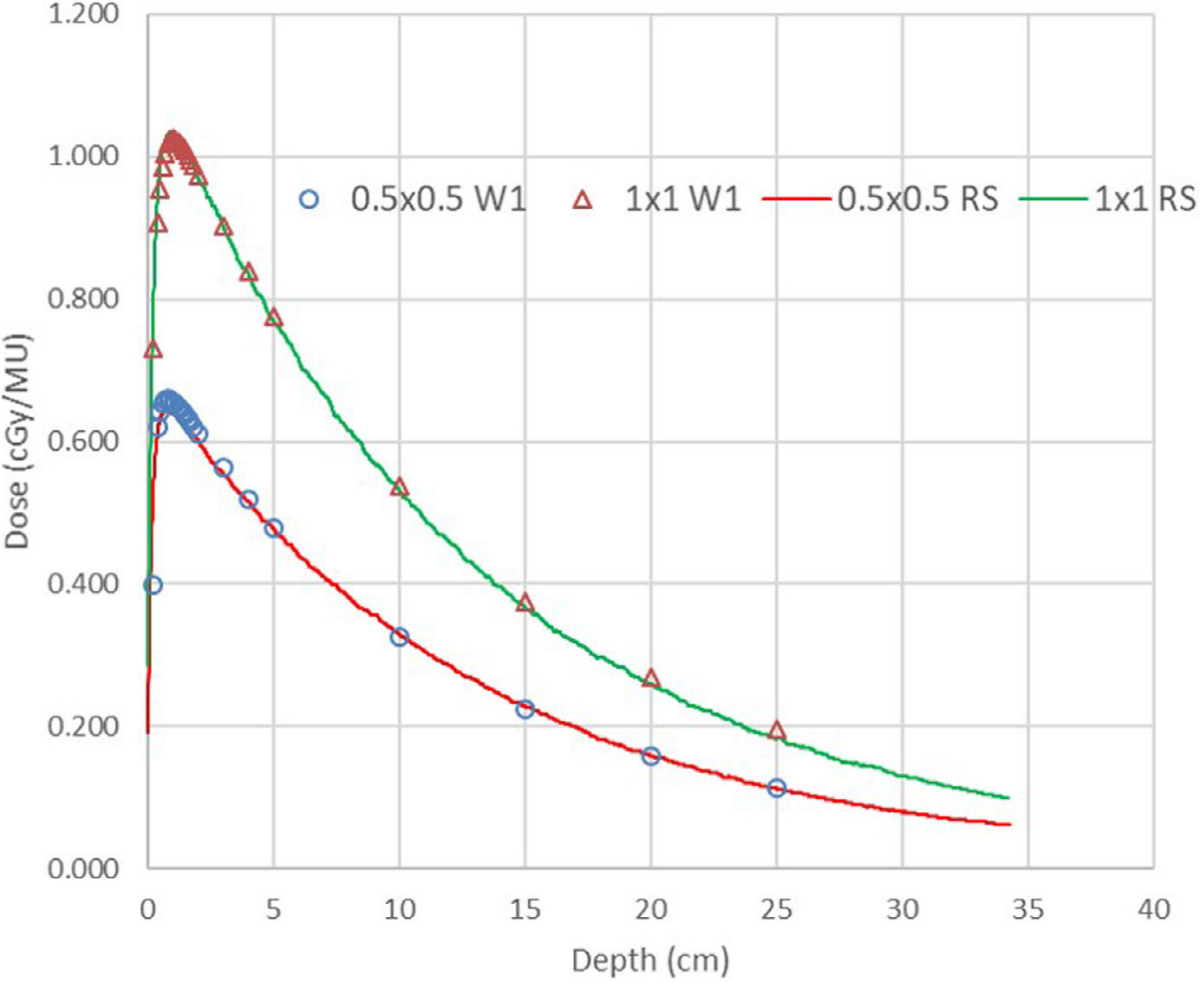
Measured (W1 scintillator) and calculated depth dose curves for the small fields (0.5 × 0.5 and 1 × 1 cm^2^).

Figure 1 helps in visualizing the agreement between the measured, calculated, and Varian standard data cross‐beam profiles. Excellent penumbra shape agreement was achieved with the primary source sizes of 0.090 and 0.075 cm in the Y and X directions, respectively. The PDD agreement between the measured and standard data observed in this work comports with the report from Nethrton et al:^2^ the 2 × 2 cm^2^ field PDD can disagree by up to one percentage point, while the rest of the curves agree much better. Figure 2 demonstrates the agreement between the calculated and measured PDDs for the smallest fields (0.5 × 0.5 and 1 × 1 cm^2^). No standard PDD data are available for comparison for those fields.

### 3.2 Relative output factors

**Fig. 3:**
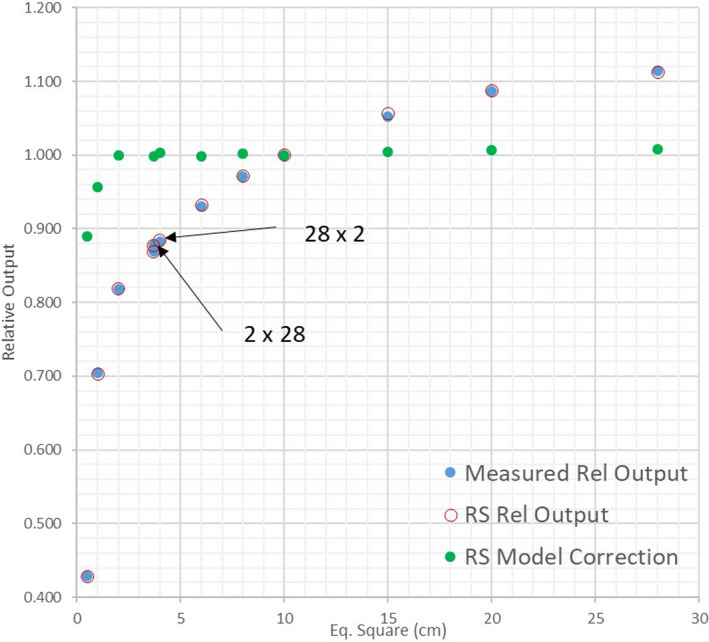
A graph of relative output factors vs field size — RayStation calculated and measured. Also shown are the beam model output correction factors.

Figure 3 shows the measured and calculated relative output factors vs field size. The maximum disagreement between the W1 scintillator and the Edge detector was 2.3% (for the smallest field) and the scintillator data were used in the TPS. The required corrections in the RayStation model are close to unity for all fields above 2 cm equivalent square, demonstrating internal consistency of the source model. The output corrections were optimized by the RayStation beam automodeling algorithm for all field sizes except 0.5 cm equivalent square. RayStation requires a symmetrical field which cannot be constructed for a 0.5 × 0.5 cm^2^ field size. It was represented in the model by an 0.333 × 1 cm^2^ symmetrical field with the same equivalent square. However, for such a narrow field the relative output is not the same as for 0.5 × 0.5 cm^2^. The output was calculated outside of the beam editor for an asymmetric field and the output correction factor was adjusted manually. As one can see in Fig. 2, this procedure leads to correct results (the depth doses are presented in dose per MU). The collimator exchange effect for orthogonal elongated fields is minimal but the small difference is correctly represented by RayStation (arrows in Fig. 3). Note that due to the depth/SSD differences the output factors are numerically different from Eclipse.

### 3.3 MLC parameters

#### 3.3.1 Tongue‐and‐Groove width

**Fig. 4:**
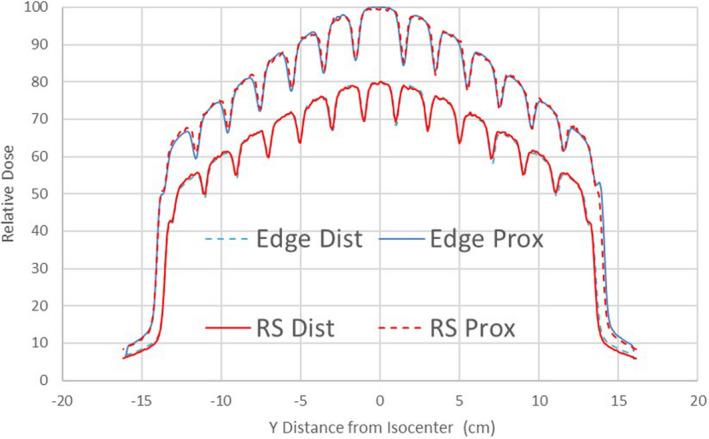
Relative diode scan dose vs RayStation calculation form the complementary bar patterns used to validate the T&G width parameter. The relative dose profiles for the distal and proximal MLC layers are presented on slightly different scales to improve visualization.

As evident from the combined complementary bars scans in Fig. 4, a typical T&G width value of 0.05 cm leads to quite good agreement with experiment and that value was fixed in the beam model. The T&G width is expected to have at most a modest effect on the final dosimetry.^16^ Also note the consistency in the Halcyon MLC gap width across the field and the ability of both the diode scan and calculation on a 1 mm grid to resolve the dose “bump” at the outer edge of the leaf resulting from the reduced thickness.

### 3.4 The MLC *Gain* parameter

**Fig. 5:**
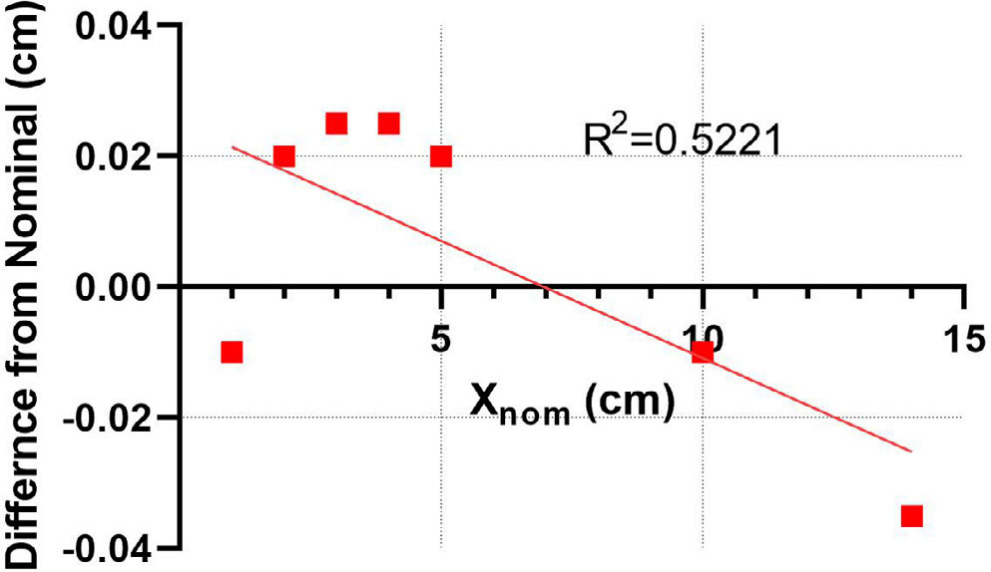
Linear fit to the experimental $\Delta X_{\mathrm{MP}}$ values as a function of X*_nom._*

The differences between the actual and nominal mid‐point positions of the two opposing leaves planned for the same position *X_nom_*, or $\Delta X_{\mathrm{MP}}$, are presented in Fig. 5 as a function of *X_nom_*. Analysis of the simple linear fit shows that the slope of the regression line is not statistically significantly different from zero (*P* = 0.07).^30^ We attributed the observed variations to measurement error^14^ and set the *Gain* value in the model at 0.0.

### 3.5 The *Offset* and *Curvature* parameters

**Fig. 6:**
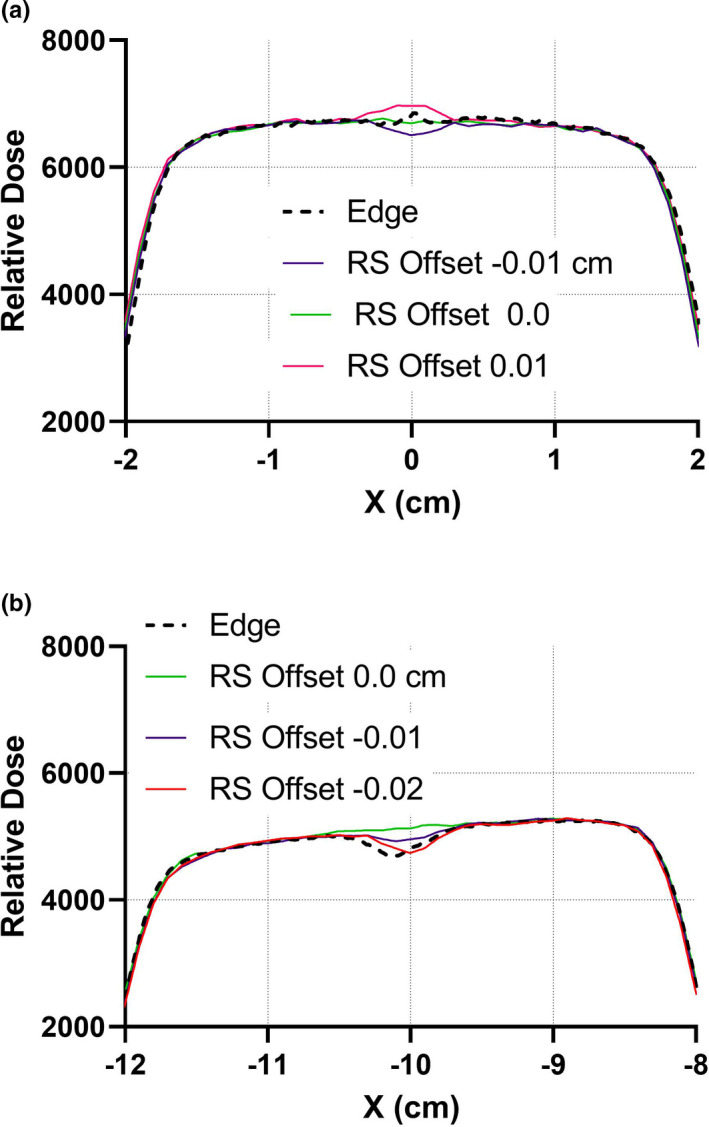
Measured (Edge) and calculated summed profiles through two abutting fields at the central axis (A) and 10 cm off‐axis (B). The off‐axis profiles are average between ±10 cm displacements and shown at −10 cm.

**Fig. 7:**
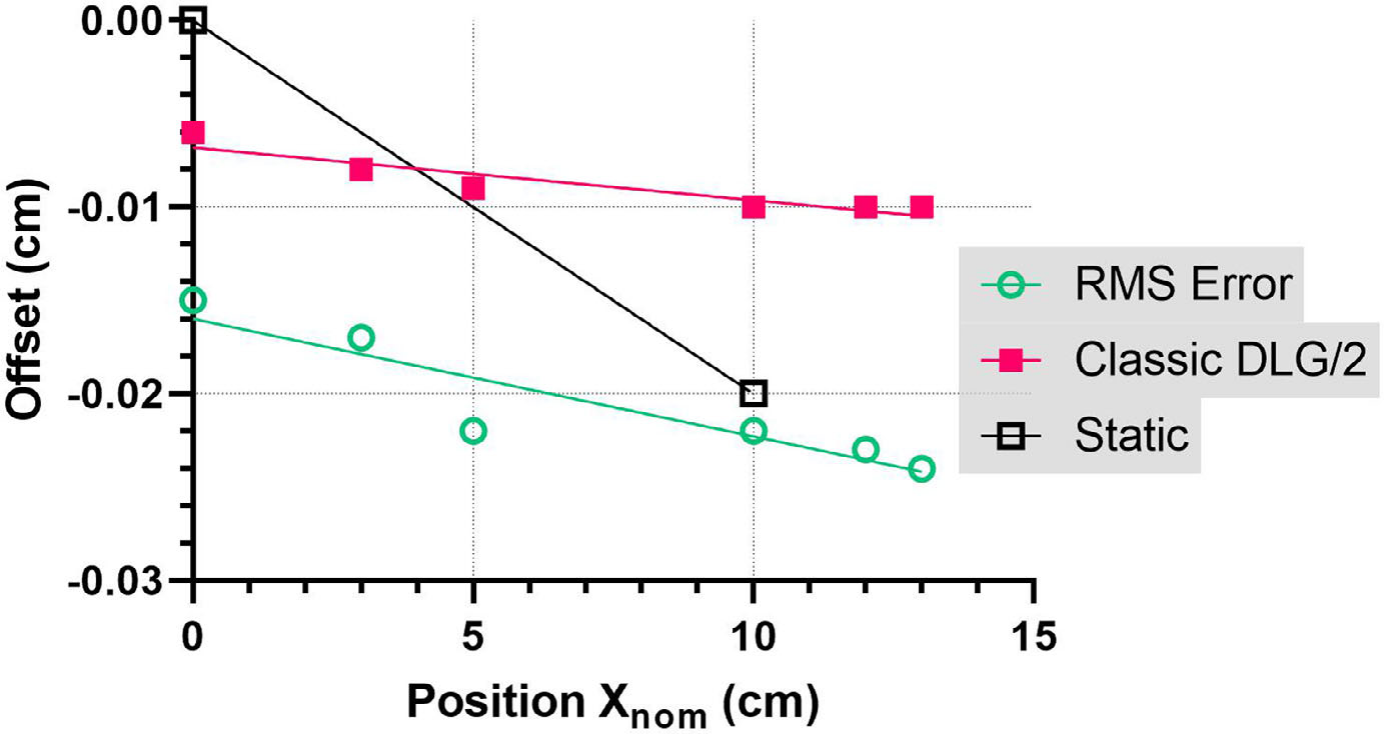
MLC offset measured by different methods vs distance from central axis. The DLG/2 values are shown as measured, while the RMS and static offset values are the optimized ones.

The leaf offset is one of the most important parameters influencing the final IMRT/VMAT results.^16^ From the static abutting field measurements (Fig. 6) the optimal $\Delta X_{D}$parameter should be 0.0 cm on the central axis and about −0.02 cm 10 cm away from it. On the other hand, dynamic gap measurements with two different analysis methods show slightly different results. The findings from all three methods are summarized in Fig. 7. Even though the optimized RMS disagreement between the RayStation calculations and IC measurements was relatively low (1.1%–1.6%, depending on the X position), the offset values obtained by simplified model exercises may not be always optimal for realistic dynamic plans.^3^, ^31^ The −0.015 cm offset value from the RMS method was chosen as an initial guess with the understanding that it would likely change based on the IMRT/VMAT tests. The *Curvature* parameter should be employed with caution as the strong quadratic function could lead to significant additional offsets away from the central axis.^14^ It was left a 0.0 cm^‐1^, with the idea of possible adjustment by experimentation on the modulated plans. The quadratic polynomial fit to the optimized offset data suggested a trial value of −4.10^‐5^ cm^‐1^, but this magnitude is likely to be at the experimental error level.^14^

### 3.6 The leaf tip width parameter

Unlike with the Millennium MLC (leaf end radius 8 cm), varying the MLC tip width by 0.1 cm did not noticeably affect the shape of the calculated abutting field profile. The flat profile obtained with *Offset* = 0.0 cm remained unchanged beyond statistical noise between leaf tip widths of 0.0 and 0.1 cm. This is in stark contrast to *Offset*, where a 0.01 cm change is readily apparent (Fig. 6). Even with the Millennium MLC, the leaf the tip width parameter in RayStation should have only a moderate effect on the resulting dosimetric agreement.^16^ It is expected to be even smaller for a taller Halcyon leaf with a flatter end. Absent evidence to the contrary, the leaf tip width was fixed at 0.0 cm to minimize the number of variables.

### 3.7 IMRT/VMAT Tests

#### 3.7.1 Ion chamber measurements

**Fig. 8:**
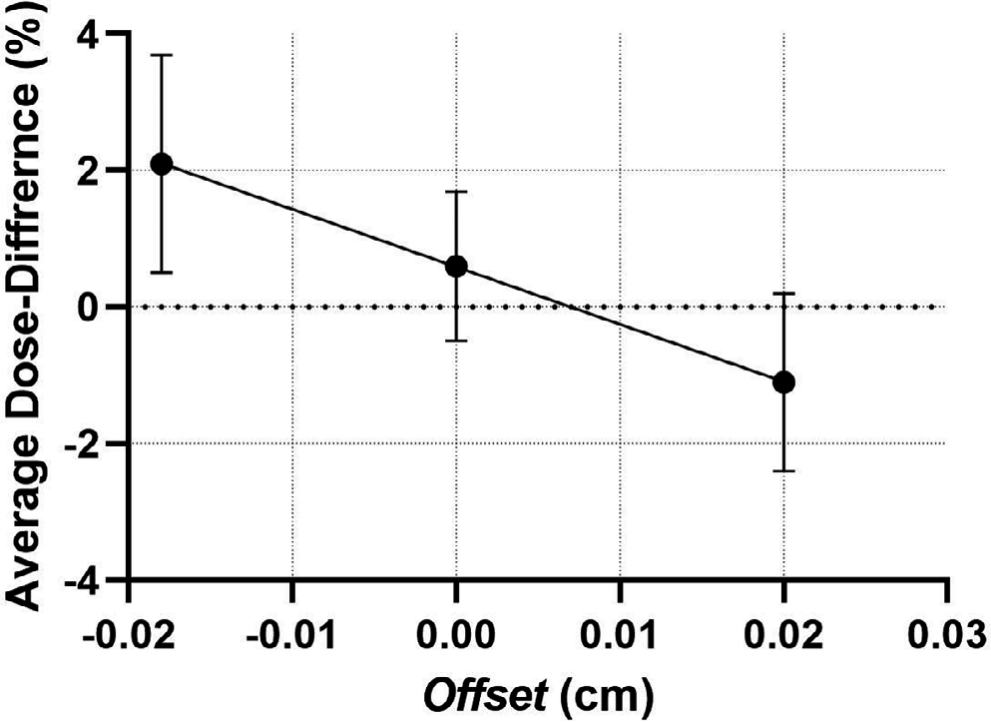
Average IC dose difference vs the *Offset* parameter. Vertical bars show one standard deviation.

The first step was to optimize the *Offset* parameter to minimize the mean difference between the measured and calculated point doses. The graph of the average dose difference across 30 measurements with three different planning techniques, as a function of the *Offset* value, is presented in Fig. 8. The X‐intercept of the linear fit resulted in the optimized *Offset* value of +0.007 cm. Keeping the *Gain* and *Curvature* values at 0, the agreement for 30 IC measurements was analyzed closely. The detailed results are tabulated in the Appendix. The overall mean value was 0.0 ± 1.1% (1SD). For 29 of 30 points the locally normalized difference between the calculated and measured dose did not exceed 1.7%. One point in the high gradient region (CShape OAR) exhibited a 4% deviation (which is equivalent to 1.5% with the global dose‐error normalization originally reported in TG119^19^ and often used since then). Varying the *Gain* and *Curvature* parameters within the limits suggested by the dynamic and static MLC offset data neither changed the average agreement beyond 0.1% nor reduced the standard deviation. Those parameters were again left at 0.0 pending further analysis.

With the plans recalculated with Eclipse, the overall mean difference between the TPS and measured dose was 1.5% ± 2.0%. This average was clearly skewed by the large locally normalized deviations (~8%) in the low‐dose high‐gradient region (C‐Shape OAR) for two plans. Excluding those would lead to the average value of 1.0% ± 1.0%. Once again, with global normalization those 8% errors would be considered on the order of 3%. Six measurement points exceeded ±2% error with Eclipse compared to one with RayStation.

#### 3.7.2 Diode array measurements

**Fig. 9:**
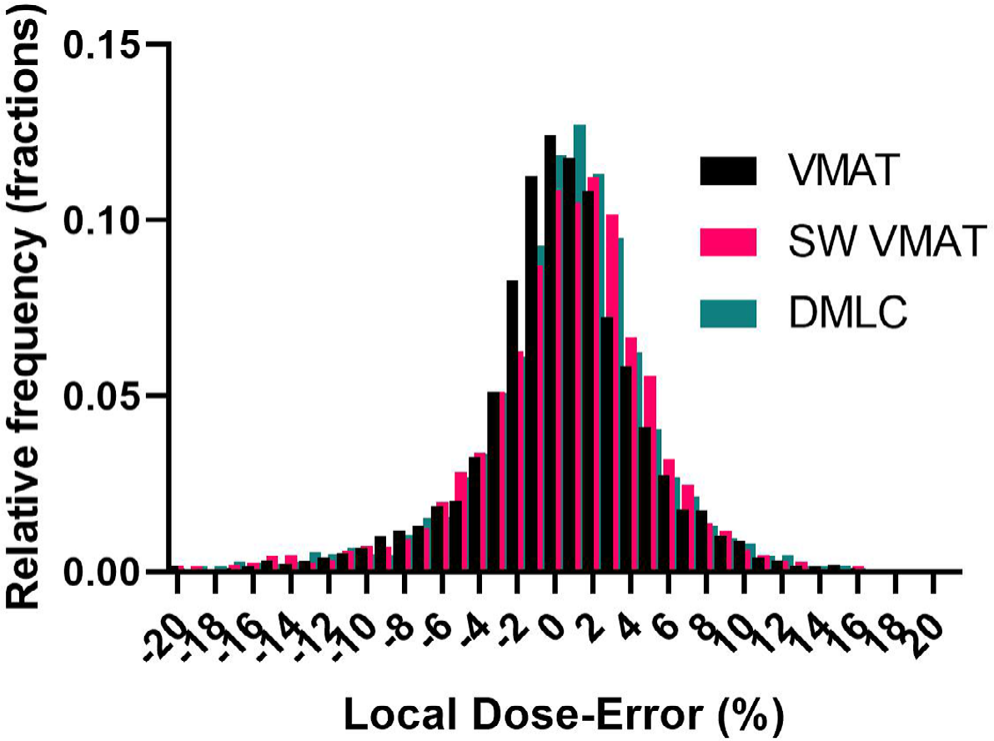
Frequency distribution of local dose errors stratified by planning technique.

Detailed gamma analysis results can be seen in the Appendix. While the passing rates with the standard TG‐218^25^ criteria are universally above 98%, the results with the 2%L/2 mm criteria show some potential differences between the planning techniques. A nonparametric ANOVA‐type test followed by multiple comparisons (Friedman test)^30^ applied to the 2%L/2 mm gamma analysis passing rates suggests statistically significant difference in median values (paired Friedman test *P* = 0.039). In pair‐wise comparisons only the difference between VMAT and SW VMAT was statistically significant (Friedman test *P* = 0.034). The median and mean locally normalized dose errors across all diodes receiving at least 10% of the maximum dose are 0.7 and 0.2 ± 5.3%, respectively. The direction of the mean and median ArcCHECK dose error (Appendix) suggests that adding a negative *Curvature* term to the MLC offset equation (i.e., decreasing the calculated dose) would pull them further away from zero. It was verified that the change decreased the gamma analysis passing rates for the two most modulated plans with large targets — Anal and H&N. A positive curvature would be contrary to the observed experimental trends consistent across all analysis methods (Fig. 7). Hence the *Curvature* coefficient was assigned the final value of 0.0. Adding a *Gain* parameter corresponding to the slope of the linear regression line (−0.0004) in Fig. 5 did not change the results beyond statistical uncertainty. Finally, the frequency distributions of dose errors grouped by planning technique are presented in Fig. 9.

ANOVA analysis^30^ of the differences of the mean of the dose‐error distributions per treatment technique found no statistically significant differences between VMAT, SW VMAT, and DMLC mean values.

#### 3.7.3 Independent patient‐specific dose verification method

First, the point doses for all plans were recalculated with the PF dose engine (DoseCHECK) preserving all calculation parameters. That gave us three sets of 30 repeated measured data points: from RayStation with a custom beam model, Eclipse, and DoseCHECK, the latter two with their standard models. The results for PF Fraction 0 (based on log file MLC positions) and DoseCHECK (based on the initial plan) were practically indistinguishable and only the DoseCHECK results are reported for brevity. The one‐way ANOVA test^30^ indicated statistically significant differences (*P* < 0.0001) between the mean dose‐error values (0.0 ± 1.1%, 1.5 ± 2.0%, and −0.6 ± 1.5% for RayStation, Eclipse, and DoseCHECK, respectively). The follow‐up Holm–Sidak’s multiple comparisons test^30^ showed that all pair‐wise differences were also statistically significant (*P* ≤ 0.007).

In clinical practice, the RayStation calculated dose would normally be compared to the PF dose reconstruction by gamma analysis. With the standard 3%G/2 mm criteria and 10% cutoff threshold, the average passing rate for 15 plans was 99.9% ± 0.1% (range 99.8%–100%). Tightening the criteria to 2%L/2 mm resulted in the average passing rate of 98.5 ± 0.8% (range 97.1%–100%). The mean local dose difference among all voxels receiving above 10% of maximum dose across all plans was 0.2% ± 0.8% (range among individual plans from −1.6% to 1.6%).

## 4 DISCUSSION

Historically, the process of commissioning a radiotherapy treatment unit included local beam data collection and beam modeling in the TPS which may or may not have been supplied by the same vendor. This approach has its drawbacks as highlighted by a number of publications from IROC Houston^16^, ^32^, ^33^, ^34^ indicating a nontrivial failure rate for the end‐to‐end hidden target dosimetric tests on the anthropomorphic phantoms. A substantial proportion of failures can be traced back to suboptimal beam modeling in the TPS.^33^ Thus an alternative approach was developed, most recently implemented for example on the Varian Halcyon^2^ and ViewRay MRIdian^35^, ^36^ platforms. Those are preconfigured, closed systems, and the end user’s role in commissioning is largely reduced to validating manufacturer’s specifications. This leads to a single set of TPS dosimetric parameters, as opposed to wide variation observed with open systems.^15^ Combined with tight manufacturing specifications, such approach ensures that minimum quality standards are met across the user population and the first published results indicate encouraging results with the IROC end‐to‐end tests.^2^, ^36^ However this approach also has its drawbacks. While having deep knowledge of their system, the vendor team may not be exposed to the full variety of clinical scenarios. A set of TPS parameters optimal for one set of clinical plans may not necessarily be the best choice for other types.^31^ A local physicist might add experimental data that would make the modeling dataset more robust. Finally, for various logistical reasons a clinic may find it beneficial to rely on a TPS of their choice to avoid the need for multiple accelerator‐specific systems. All of the above considerations were taken into account in our decision to commission an independent vendor TPS for the newly installed Halcyon accelerator.

We have made the beam dataset used for modeling more complete, by adding dose profiles scanned with both a diode and an ion chamber, and also provided relative output factors for very small fields, down to 0.5 × 0.5 cm^2^. The former gives a choice of a best tool for the job in terms of modeling the penumbra vs the inner portion of the beam. The latter is beneficial in optimizing dosimetric accuracy of small MLC apertures found in highly modulated plans.

Our attempts to optimize the all‐important leaf‐end shape and offset parameters agree with various previous findings that the results based on simple static and dynamic fields depend on the measurement techniques and conditions^2^, ^3^, ^37^ and ultimately highly modulated realistic plans are necessary to hone those values.^13^, ^38^, ^39^ Of all variables in the RayStation model, we find the leaf tip width to be the least intuitive. It is an incremental attempt at modeling a rounded leaf end, in between the Eclipse flat end approach with a constant offset across the field^40^ and Pinnacle ray‐tracing through the rounded leaf end shaped and positioned almost as in the real world.^41^ We failed to find a quantitative relationship between the optimal leaf tip width value and the physical leaf shape for various MLC models. On a practical level, our static field experiments indicated that this parameter had no appreciable effect on the Halcyon penumbra profiles, and it was left at 0. While the MLC *Offset* parameter was eventually optimized at +0.007 cm, the model‐based *Gain* and *Curvature* values were so close to zero that the difference was attributed to experimental uncertainties.^14^ Our optimal *Offset* value is close to the standard Eclipse DLG/2^40^ value of +0.005 cm. This is reasonable since with the leaf tip width set at 0, the leaf end properties in RayStation correspond to a flat tip. Since all the leaves have the same width, it is assumed the offset parameters do not change along the Y direction, unlike with the variable leaf width Millennium MLCs.^40^

It would be interesting to see the authors of the recent paper on comprehensive model‐based MLC commissioning in RayStation^14^ expand their work to the Halcyon MLC, providing the optimized value for the leaf tip width among other parameters. Their approach is very efficient compared to the traditional trial and error solutions.

In the meantime, multiple ion chamber measurements in a phantom still remain the best predictor of the TPS dosimetric commissioning quality,^20^, ^42^, ^43^ and consequently we made the final modeling choices based on the results of 30 measurements for 15 VMAT and sliding window IMRT plans analyzed with stringent local dose‐error normalization. Many of the plans were highly modulated, requiring up to 10 MU per 1 cGy of target dose, compared to the maximum of 6 MU/cGy in the model parameters analysis by Glenn et al.^16^ Based on the ion chamber data, our optimized RayStation Halcyon beam model works well for all three planning techniques. For 96.6% of the measurement points the local dose error did not exceed ±1.7%. That included low‐dose points, while 3% agreement was previously considered satisfactory for predominantly high‐dose locations.^43^ The statistical 99% confidence level of the mean dose error (0.0%) was calculated at ±0.5%. The TG‐119‐style 95% confidence limit (mean ± 1.96SD) ^19^ is from −2.1 to + 2.1%. While detailed comparisons are hindered by different plans used for end‐to‐end testing, grossly our results compare favorably with the two‐institution^2^ and single‐institution^1^ reports, both using the preconfigured Eclipse TPS. The average IC‐measured minus TPS dose errors are 0.0 ± 1.1% for RayStation in this work, −0.9 ± 1.1% by Netherton et al.,^2^ and 0.8 ± 1.4% by Laugeman et al.^1^ The MU per cGy of prescribed dose range was reported in the latter study (2–9, average 4.5) and it is roughly comparable to ours (1.5−10, average 6). After eliminating two high‐gradient outliers (discussed previously), our mean dose error for the plans recalculated in Eclipse with the Acuros algorithm was 1.0% ± 1.1%, more in line with Laugman et al.^1^ than Netherton et al.^2^ in terms of the error direction. However, the differences are sufficiently small for all results to be considered acceptable. Somewhat larger point dose errors with Eclipse were reported by De Rover et al.^4^

All our ArcCHECK results exceed the standard TG‐218 recommendations, that is, gamma analysis passing rates ≥95% for the 3%G/2 mm/10% criteria combination.^25^ The average passing rate was 99.3 ± 0.5%, generally comparing favorably to 99.1 ± 0.9% with Eclipse in Ref. [1] By the TG‐119 methodology, the 95% confidence limit was ≥98.3% passing rate with the 3%G/2mm criteria.

The average ArcCHECK 2%L/2 mm passing rates varied between different planning techniques and there was enough statistical power in pair‐wise post‐ANOVA multiple comparisons to ascertain that at least the difference between VMAT and SW VMAT was statistically significant. The average passing rates for VMAT, IMRT, and SW VMAT (93.0 ± 2.3%, 91.1 ± 2.8%, and 88.2 ± 3.6%, respectively) are stratified exactly opposite to the increasing order of average MU used (848, 1274, and 1466, respectively), but this correlation is not statistically significant.

Perhaps the most important finding from the ArcCHECK experiments is that the average of dose errors from a large number of point detectors (581–1009 per delivery) is close to zero: 0.1 ± 5.0%, 0.0 ± 5.7%, and 0.3 ± 5.3% for VMAT, IMRT, and SW VMAT respectively. The t test indicates that only the SW VMAT mean deviation is significantly different from 0 (*P* = 0.0007), in line with the lowest gamma analysis passing rates. The standard deviations are driven up by the detectors in the high gradient areas, but the minimal bias in the mean values comports with the IC measurements and is consistent with a well‐commissioned beam model. Since the ArcCHECK detectors are positioned 10.5 cm away from the central axis, these results additionally validate the choice to leave the MLC *Gain* and *Curvature* parameters at zero.

Kerns et al.^32^ have pointed out the value of recalculation with an independent TPS, based on some class‐specific standard beam data, as a quality assurance tool for beam model commissioning. Agreement with both Eclipse and DoseCHECK boosted our confidence in the beam model.

The Halcyon/RayStation clinical workflow is quite similar to other combinations of linacs and TPSs from different vendors. The required data are pushed from the RayStation to the Aria DICOM receiver. Those include the planning CT dataset and DICOM RT Plan, Dose (composite) and Structure objects. The number of fractions is automatically lifted from the RT Plan and cannot be manually changed in Aria. The two minor practical details include the need to copy a transferred RayStation plan inside the Aria R&V system to make it compliant with certain internal checks, and to have a predefined cone beam CT field as a part of the original plan. The latter is accomplished by a simple script provided by the TPS vendor. Currently the MV CBCT or MV imaging cannot be added through that script, which results in the only loss of functionality compared to the original system.

While this work is primarily devoted to dosimetric accuracy, an important part of using an independent TPS with the Halcyon is safety considerations. We would like to emphasize that the safety/consistency checks built in the Aria/Halcyon system are in no way circumvented and are occasionally duplicated. RayStation explicitly checks that any plan cannot have more than two isocenters, spaced in compliance with the Halcyon 2.0 rules. A potential bore collision test is also included in the script. Upon transmission to Aria, the plan undergoes the same validation checks as would any Eclipse plan, called Planning Approval and Treatment Approval. That includes, among other things, the checks for the maximum number of isocenters per plan (2) and for potential collision with the bore. To facilitate collision avoidance, it is required that the external patient contour and support structure (couch) are present in the dataset, the latter is at least as long as the patient CT dataset, and both are fully covered by the calculation grid. We tested all these features by intentionally supplying plans that violated the rules one at a time. The source‐to‐surface distances are recomputed by Aria and presented side‐by‐side with the planned values. The dose prescription and fractionation are also checked prior to scheduling. The fidelity of dosimetry‐related DICOM data transfer is implicit in the dosimetric commissioning results. In addition, we verified with phantom CBCT that the plan‐based shifts between the localization point and the isocenter were correct in four cardinal patient orientations. Finally, the accelerator software and hardware collision avoidance mechanisms function independently of the treatment plan.

## 5 CONCLUSIONS

The self‐contained Halcyon radiotherapy platform was successfully “opened up” for planning and patient‐specific dose verification with independent systems, consolidating resources and allowing use of the same tools and workflows as for the rest of the Varian machines in the department. The planning dose accuracy is at least on par with the dedicated preconfigured TPS. Since the plans are routed through Aria R&V system, they undergo the same safety and consistency checks as would any plan generated in Eclipse. Given the expected similarity between the Halcyon machines’ radiation output, the beam model parameters presented in this work should provide at least a solid first approximation for other users interested in pursuing a similar route.

## Author’s contribution

Amarjit Saini, Chris Tichacek, William Johansson, and Gage Redler were involved in design and execution of portions of experimental work, and manuscript revision and approval. Geoffrey Zhang was involved in design of portions of experimental work, and manuscript revision and approval. Eduardo G. Moros was involved in contribution to the overall conceptual design, and manuscript editing and approval. Muqeem Qayyum was involved in design of the experimental approach and data collection, results evaluation, and manuscript preparation and approval. Vladimir Feygelman was involved in overall conceptual design, design and execution of portions of experimental work, and manuscript writing and approval.

## Conflict of interest

Muqeem Qayyum is an employee of RaySearch. No other potential conflict of interest is reported.

## Appendix

Table 1 provides details of the measurement setup and instrumentation for the basic beam data collection (Section II.B.2).

**Table 1:** Beam data measurements and instrumentation. FS = Field Size.

Quantity	Purpose	Setup	Instrumentation/Method
PDD	Basic beam modeling	90 cm SSD. FS 15 × 15 to 28 × 28 cm^2^	Continuous scanning in the Blue Phantom (IBA, Schwartzenbrook, Germany). IBA CC13 ion chamber
		FS 2 × 2 to 10 × 10 cm^2^	Edge diode detector (Sun Nuclear Corp., Melbourne, FL)
	Small field data input	FS 0.5 × 0.5 & 1 × 1 cm^2^	W1 Scintillator with Supermax electrometer (Standard Imaging, Middleton, WI). Step‐by‐step integration.
Cross beam profiles	Basic beam modeling	FS 20 × 20 to 28 × 28 cm^2^	CC13
		FS 2 × 2 to 10 × 10 cm^2^	Edge
	MLC modeling	FS 2x2 to 28 × 28 cm^2^	Edge
Relative output factors	Output correction factors in beam model	90 cm SSD, 10 cm depth. FS 4 × 4 to 28 × 28 cm^2^	Water tank, CC13
		FS 0.5 × 0.5 to 4 × 4 cm^2^	W1, confirmed with Edge

Table 2 presents the detailed results of the IMRT/VMAT dosimetric verification measurements (Section 2.2.2, 3.7.G.).

**Table 2:** Ion Chamber dose agreement, ArcCHECK gamma analysis and median/mean dose differences. All dose differences reported as Measured minus RayStation Calculated.

Plan	ΔD Meas. ‐TPS (%)		γ pass rate (%)					
	P High	P low	3%G/2 mm	3%L/2 mm	2%L/2 mm	Median ΔD (%)	Ave ΔD (%)	MU
Cshape VMAT	‐0.4%	0.0%	99.5	93.8	91.1	0.2	‐0.2	1388
ABD VMAT	‐0.1%	0.8%	100.0	98.2	96.3	‐0.4	‐0.1	358
ANAL VMAT	0.4%	0.5%	99.7	93.5	92.4	0.3	‐0.5	1257
HN VMAT	0.5%	‐1.7%	99.9	94.1	90.9	0.9	0.7	629
ProstBed VMAT	‐0.1%	0.9%	99.7	96.8	94.4	0.6	0.5	610
Ave	0.1%	0.1%	99.8	95.3	93.0	0.3	0.1	848
SD	0.4%	1.1%	0.2	2.1	2.3	0.5	0.5	
Cshape SW VMAT	0.5%	0.0%	99.1	91.1	89.7	0.4	‐0.3	1844
ABD SW VMAT	0.7%	‐1.0%	99.1	93.0	90.2	0.7	0.3	1075
ANAL SW VMAT	0.6%	0.5%	98.9	92.5	89.6	0.3	‐0.3	1183
HN SW VMAT	0.2%	1.0%	98.2	87.2	81.8	1.2	0.8	1655
ProstBed SW VMAT	0.1%	1.4%	99.8	93.5	89.6	1.2	0.8	1574
Ave	0.4%	0.4%	99.0	91.5	88.2	0.8	0.3	1466
SD	0.2%	1.0%	0.6	2.5	3.6	0.4	0.6	
Cshape DMLC	‐0.7%	‐4.0%	98.6	95.9	94.3	0.3	‐0.4	1151
ABD DMLC	0.6%	‐1.2%	99.0	93.7	88.8	0.9	0.6	1073
ANAL DMLC	0.8%	‐0.1%	98.6	94.3	92.2	‐0.2	‐0.6	1513
HN DMLC	0.7%	‐0.5%	99.3	92.8	87.6	1.6	0.8	1243
ProstBed DMLC	‐1.3%	0.6%	99.7	95.9	92.6	0.4	‐0.5	1389
Ave	0.0%	‐1.0%	99.0	94.5	91.1	0.6	0.0	1273
SD	1.0%	1.8%	0.5	1.4	2.8	0.7	0.7	
Overall Ave	0.2%	‐0.2%	99.3	93.8	90.8	0.6	0.1	
SD	0.6%	1.4%	0.5	2.6	3.4	0.5	0.5	
IC Ave High and Low	0.0%							
SD	1.1%							
